# Bis[1-ethyl-6-fluoro-7-(4-methyl­piperazin-1-yl)-4-oxo-1,4-dihydro­quinoline-3-carboxyl­ato-κ^2^ *O* ^3^,*O* ^4^]­copper(II)

**DOI:** 10.1107/S1600536809003584

**Published:** 2009-02-04

**Authors:** Wei Qi, Jing Gao, Di Liang, Zhe An

**Affiliations:** a School of Pharmaceutical Science, Harbin Medical University, Harbin 150086, People’s Republic of China; b Department of Pharmacy, Mudanjiang Medical University, Mudanjiang 157011, People’s Republic of China

## Abstract

In the title compound, [Cu(C_17_H_19_FN_3_O_3_)_2_], the Cu^II^ atom (site symmetry 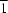) exhibits a slightly distorted CuO_4_ square-planar geometry defined by two bidentate *O*,*O*′-bonded 1-ethyl-6-fluoro-7-(4-methyl­piperazin-1-yl)-4-oxo-1,4-dihydro­quinoline-3-carboxyl­ate (perfloxacinate) anions.

## Related literature

For the silver, manganese, cobalt and zinc complexes of the perfloxacinate (pef) anion, see: Baenziger *et al.* (1986 ▶); An, Huang & Qi (2007 ▶); An, Qi & Huang (2007 ▶); Qi *et al.*(2008 ▶), respectively. For background on the medicinal uses of Hpef, see: Mizuki *et al.* (1996 ▶).
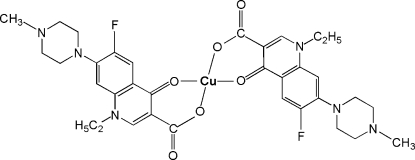


## Experimental

### 

#### Crystal data

[Cu(C_17_H_19_FN_3_O_3_)_2_]
*M* *_r_* = 728.24
Triclinic, 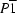

*a* = 8.5548 (17) Å
*b* = 10.253 (2) Å
*c* = 10.467 (2) Å
α = 95.22 (3)°
β = 109.63 (3)°
γ = 108.01 (3)°
*V* = 802.7 (4) Å^3^
*Z* = 1
Mo *K*α radiation
μ = 0.75 mm^−1^
*T* = 296 (2) K
0.36 × 0.28 × 0.19 mm

#### Data collection

Bruker SMART CCD area-detector diffractometer
Absorption correction: multi-scan (*SADABS*; Bruker, 2001 ▶) *T*_min_ = 0.774, *T*_max_ = 0.871
7880 measured reflections
3633 independent reflections
3274 reflections with *I* > 2σ(*I*)
*R*_int_ = 0.022

#### Refinement

*R*[*F* ^2^ > 2σ(*F* ^2^)] = 0.033
*wR*(*F* ^2^) = 0.103
*S* = 1.14
3633 reflections
225 parameters
H-atom parameters constrained
Δρ_max_ = 0.35 e Å^−3^
Δρ_min_ = −0.37 e Å^−3^

### 

Data collection: *SMART* (Bruker, 1998 ▶); cell refinement: *SAINT* (Bruker, 1998 ▶); data reduction: *SAINT*; program(s) used to solve structure: *SHELXS97* (Sheldrick, 2008 ▶); program(s) used to refine structure: *SHELXL97* (Sheldrick, 2008 ▶); molecular graphics: *SHELXTL* (Sheldrick, 2008 ▶); software used to prepare material for publication: *SHELXTL*.

## Supplementary Material

Crystal structure: contains datablocks I, global. DOI: 10.1107/S1600536809003584/hb2904sup1.cif

Structure factors: contains datablocks I. DOI: 10.1107/S1600536809003584/hb2904Isup2.hkl

Additional supplementary materials:  crystallographic information; 3D view; checkCIF report

## Figures and Tables

**Table 1:** Selected bond lengths (Å)

Cu1—O1	1.8858 (15)
Cu1—O3	1.9247 (13)

**Fig. 1.**
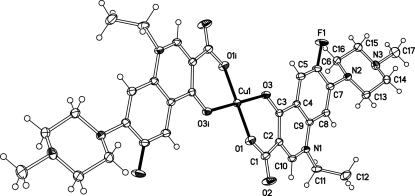
The molecular structure of (I), show the Cu coordination, showing 50% displacement ellipsoids (arbitrary spheres for the H atoms). [Symmetry code: (i) 1–x, 1–y, 1–z.]

## supplementary crystallographic information

### Comment

Pefloxacin (Hpef, C_17_H_20_FN_3_O_3_, 1-ethyl-6-fluoro-7-(4-methylpiperazin-1-yl)-4-oxo-quinoline -3-carboxylic acid) is member of a class of quinolones used to treat infections (Mizuki *et al.*, 1996;). The silver(I), manganese(II), cobalt(II) and zinc(II) derivative of the pefloxacinate (pef) anion has been reported (Baenziger *et al.*,1986; An, Huang & Qi (2007); An, Qi & Huang (2007); Qi *et al.*(2008); Qi *et al.*, 2008). The title copper(II)-containing complex of pef, (I), is reported here.

The structure of (I) is built up from Cu^2+^ cations (site symmetry 1) anions (pef) ligands, (Fig. 1). It is confirmed that four coordinating O atoms arround Cu^II^ cation form a square planar configuration. (Table 1).

### Experimental

A mixture of Cu(CH_3_COO)_2_.H_2_O (0.050 g, 0.25 mmol), Hpef (0.17 g, 0.5 mmol) and water (12 ml) was stirred for 30 min in air. The mixture was then transferred to a 23 ml Teflon-lined hydrothermal bomb. The bomb was kept at 433 K for 72 h under autogenous pressure. Upon cooling, blue prisms of (I) were obtained from the reaction mixture.

### Refinement

All H atoms on C atoms were generated geometrically and refined as riding atoms with C—H= 0.93–0.97Å and *U*_iso_(H)= 1.2*U*_eq_(C) or 1.5U_eq_(methyl C).

### Crystal data

**Table:**

[Cu(C_17_H_19_FN_3_O_3_)_2_]	*Z* = 1
*M**_r_* = 728.24	*F*(000) = 379
Triclinic, *P*1	*D*_x_ = 1.506 Mg m^−^^3^
Hall symbol: -P 1	Mo *K*α radiation, λ = 0.71073 Å
*a* = 8.5548 (17) Å	Cell parameters from 7808 reflections
*b* = 10.253 (2) Å	θ = 3.1–27.5°
*c* = 10.467 (2) Å	µ = 0.75 mm^−^^1^
α = 95.22 (3)°	*T* = 296 K
β = 109.63 (3)°	Prism, blue
γ = 108.01 (3)°	0.36 × 0.28 × 0.19 mm
*V* = 802.7 (4) Å^3^	

### Data collection

**Table:**

Bruker SMART CCD area-detector diffractometer	3633 independent reflections
Radiation source: fine-focus sealed tube	3274 reflections with *I* > 2σ(*I*)
graphite	*R*_int_ = 0.022
Detector resolution: 0 pixels mm^-1^	θ_max_ = 27.5°, θ_min_ = 3.1°
φ and ω scans	*h* = −11→10
Absorption correction: multi-scan (*SADABS*; Bruker, 2001)	*k* = −10→13
*T*_min_ = 0.774, *T*_max_ = 0.871	*l* = −13→13
7880 measured reflections	

### Refinement

**Table:**

Refinement on *F*^2^	Primary atom site location: structure-invariant direct methods
Least-squares matrix: full	Secondary atom site location: difference Fourier map
*R*[*F*^2^ > 2σ(*F*^2^)] = 0.033	Hydrogen site location: inferred from neighbouring sites
*wR*(*F*^2^) = 0.103	H-atom parameters constrained
*S* = 1.14	*w* = 1/[σ^2^(*F*_o_^2^) + (0.06*P*)^2^ + 0.1742*P*] where *P* = (*F*_o_^2^ + 2*F*_c_^2^)/3
3633 reflections	(Δ/σ)_max_ < 0.001
225 parameters	Δρ_max_ = 0.35 e Å^−^^3^
0 restraints	Δρ_min_ = −0.37 e Å^−^^3^

### Special details

**Table:**

Geometry. All e.s.d.'s (except the e.s.d. in the dihedral angle between two l.s. planes) are estimated using the full covariance matrix. The cell e.s.d.'s are taken into account individually in the estimation of e.s.d.'s in distances, angles and torsion angles; correlations between e.s.d.'s in cell parameters are only used when they are defined by crystal symmetry. An approximate (isotropic) treatment of cell e.s.d.'s is used for estimating e.s.d.'s involving l.s. planes.
Refinement. Refinement of *F*^2^ against ALL reflections. The weighted *R*-factor *wR* and goodness of fit *S* are based on *F*^2^, conventional *R*-factors *R* are based on *F*, with *F* set to zero for negative *F*^2^. The threshold expression of *F*^2^ > σ(*F*^2^) is used only for calculating *R*-factors(gt) *etc*. and is not relevant to the choice of reflections for refinement. *R*-factors based on *F*^2^ are statistically about twice as large as those based on *F*, and *R*- factors based on ALL data will be even larger.

### Fractional atomic coordinates and isotropic or equivalent isotropic displacement parameters (Å^2^)

**Table:**

	*x*	*y*	*z*	*U*_iso_*/*U*_eq_	
Cu1	0.5000	0.5000	0.5000	0.02371 (11)	
F1	0.82563 (18)	0.16415 (13)	0.12581 (17)	0.0524 (4)	
O1	0.59169 (18)	0.69304 (14)	0.50180 (16)	0.0337 (3)	
O2	0.7906 (2)	0.89436 (16)	0.5126 (2)	0.0579 (5)	
O3	0.63511 (17)	0.45479 (13)	0.39993 (15)	0.0302 (3)	
N1	1.09849 (19)	0.72064 (15)	0.39047 (16)	0.0239 (3)	
N2	1.1346 (2)	0.33064 (16)	0.10980 (17)	0.0291 (3)	
N3	1.3578 (2)	0.24538 (19)	−0.00270 (19)	0.0353 (4)	
C1	0.7358 (3)	0.76677 (19)	0.4892 (2)	0.0294 (4)	
C2	0.8370 (2)	0.69045 (18)	0.44045 (19)	0.0244 (4)	
C3	0.7735 (2)	0.54329 (18)	0.39289 (18)	0.0231 (3)	
C4	0.8738 (2)	0.49016 (18)	0.32802 (19)	0.0233 (3)	
C5	0.8070 (2)	0.34754 (19)	0.2611 (2)	0.0297 (4)	
H5A	0.7014	0.2864	0.2617	0.036*	
C6	0.8976 (3)	0.30001 (19)	0.1959 (2)	0.0315 (4)	
C7	1.0598 (2)	0.38636 (19)	0.1906 (2)	0.0267 (4)	
C8	1.1264 (2)	0.52630 (19)	0.2581 (2)	0.0258 (4)	
H8A	1.2336	0.5861	0.2587	0.031*	
C9	1.0346 (2)	0.57967 (18)	0.32596 (18)	0.0227 (3)	
C10	0.9982 (2)	0.77176 (18)	0.43938 (19)	0.0254 (4)	
H10A	1.0400	0.8680	0.4748	0.031*	
C11	1.2746 (2)	0.81963 (19)	0.4025 (2)	0.0307 (4)	
H11A	1.3593	0.7721	0.4229	0.037*	
H11B	1.3171	0.8985	0.4797	0.037*	
C12	1.2671 (3)	0.8743 (2)	0.2716 (3)	0.0470 (6)	
H12A	1.2253	0.7967	0.1948	0.071*	
H12B	1.3839	0.9358	0.2840	0.071*	
H12C	1.1873	0.9249	0.2530	0.071*	
C13	1.2615 (3)	0.4313 (2)	0.0678 (2)	0.0326 (4)	
H13A	1.3746	0.4760	0.1459	0.039*	
H13B	1.2165	0.5036	0.0366	0.039*	
C14	1.2866 (3)	0.3524 (2)	−0.0493 (2)	0.0333 (4)	
H14A	1.1732	0.3084	−0.1272	0.040*	
H14B	1.3677	0.4179	−0.0803	0.040*	
C15	1.2292 (3)	0.1448 (2)	0.0361 (2)	0.0352 (4)	
H15A	1.2732	0.0716	0.0657	0.042*	
H15B	1.1178	0.1013	−0.0439	0.042*	
C16	1.1970 (3)	0.2168 (2)	0.1528 (2)	0.0332 (4)	
H16A	1.1085	0.1490	0.1757	0.040*	
H16B	1.3065	0.2551	0.2349	0.040*	
C17	1.3936 (4)	0.1748 (3)	−0.1120 (3)	0.0563 (7)	
H17A	1.4481	0.1096	−0.0768	0.084*	
H17B	1.4724	0.2435	−0.1407	0.084*	
H17C	1.2838	0.1252	−0.1900	0.084*	

### Atomic displacement parameters (Å^2^)

**Table:**

	*U*^11^	*U*^22^	*U*^33^	*U*^12^	*U*^13^	*U*^23^
Cu1	0.02365 (17)	0.02145 (17)	0.03097 (19)	0.00834 (12)	0.01668 (13)	0.00415 (12)
F1	0.0481 (7)	0.0230 (6)	0.0834 (10)	−0.0009 (5)	0.0416 (7)	−0.0160 (6)
O1	0.0325 (7)	0.0262 (7)	0.0559 (9)	0.0138 (5)	0.0299 (7)	0.0110 (6)
O2	0.0654 (11)	0.0217 (7)	0.1127 (16)	0.0147 (7)	0.0680 (12)	0.0092 (9)
O3	0.0283 (6)	0.0214 (6)	0.0445 (8)	0.0039 (5)	0.0246 (6)	0.0008 (6)
N1	0.0220 (7)	0.0178 (7)	0.0334 (8)	0.0060 (5)	0.0139 (6)	0.0034 (6)
N2	0.0363 (8)	0.0222 (8)	0.0393 (9)	0.0123 (6)	0.0254 (7)	0.0066 (7)
N3	0.0379 (9)	0.0391 (10)	0.0392 (10)	0.0191 (7)	0.0234 (8)	0.0049 (8)
C1	0.0347 (9)	0.0217 (9)	0.0419 (11)	0.0126 (7)	0.0242 (8)	0.0078 (8)
C2	0.0278 (8)	0.0210 (8)	0.0297 (9)	0.0104 (7)	0.0162 (7)	0.0049 (7)
C3	0.0244 (8)	0.0214 (8)	0.0263 (9)	0.0083 (6)	0.0131 (7)	0.0053 (7)
C4	0.0248 (8)	0.0192 (8)	0.0292 (9)	0.0077 (6)	0.0147 (7)	0.0048 (7)
C5	0.0291 (9)	0.0207 (9)	0.0405 (11)	0.0047 (7)	0.0200 (8)	0.0020 (8)
C6	0.0329 (9)	0.0179 (8)	0.0440 (11)	0.0041 (7)	0.0218 (8)	−0.0016 (8)
C7	0.0295 (9)	0.0224 (9)	0.0329 (9)	0.0102 (7)	0.0177 (8)	0.0037 (7)
C8	0.0247 (8)	0.0219 (8)	0.0345 (9)	0.0083 (6)	0.0162 (7)	0.0056 (7)
C9	0.0246 (8)	0.0185 (8)	0.0271 (9)	0.0088 (6)	0.0119 (7)	0.0041 (7)
C10	0.0287 (8)	0.0179 (8)	0.0319 (9)	0.0086 (7)	0.0148 (7)	0.0027 (7)
C11	0.0219 (8)	0.0205 (8)	0.0489 (12)	0.0038 (6)	0.0178 (8)	0.0007 (8)
C12	0.0486 (12)	0.0361 (12)	0.0679 (16)	0.0106 (10)	0.0388 (12)	0.0182 (11)
C13	0.0386 (10)	0.0251 (9)	0.0428 (11)	0.0110 (8)	0.0265 (9)	0.0077 (8)
C14	0.0374 (10)	0.0336 (10)	0.0347 (10)	0.0115 (8)	0.0220 (8)	0.0069 (8)
C15	0.0459 (11)	0.0307 (10)	0.0407 (11)	0.0203 (9)	0.0249 (9)	0.0079 (8)
C16	0.0454 (11)	0.0308 (10)	0.0370 (11)	0.0198 (8)	0.0258 (9)	0.0100 (8)
C17	0.0764 (18)	0.0512 (15)	0.0686 (17)	0.0307 (13)	0.0547 (15)	0.0096 (13)

### Geometric parameters (Å, °)

**Table:**

Cu1—O1^i^	1.8858 (15)	C6—C7	1.419 (3)
Cu1—O1	1.8858 (15)	C7—C8	1.387 (3)
Cu1—O3	1.9247 (13)	C8—C9	1.411 (2)
Cu1—O3^i^	1.9247 (13)	C8—H8A	0.9300
F1—C6	1.356 (2)	C10—H10A	0.9300
O1—C1	1.288 (2)	C11—C12	1.517 (3)
O2—C1	1.215 (2)	C11—H11A	0.9700
O3—C3	1.279 (2)	C11—H11B	0.9700
N1—C10	1.341 (2)	C12—H12A	0.9600
N1—C9	1.389 (2)	C12—H12B	0.9600
N1—C11	1.490 (2)	C12—H12C	0.9600
N2—C7	1.397 (2)	C13—C14	1.517 (3)
N2—C13	1.465 (2)	C13—H13A	0.9700
N2—C16	1.473 (2)	C13—H13B	0.9700
N3—C15	1.454 (3)	C14—H14A	0.9700
N3—C14	1.458 (3)	C14—H14B	0.9700
N3—C17	1.465 (3)	C15—C16	1.516 (3)
C1—C2	1.505 (2)	C15—H15A	0.9700
C2—C10	1.378 (2)	C15—H15B	0.9700
C2—C3	1.412 (2)	C16—H16A	0.9700
C3—C4	1.451 (2)	C16—H16B	0.9700
C4—C9	1.406 (2)	C17—H17A	0.9600
C4—C5	1.408 (3)	C17—H17B	0.9600
C5—C6	1.354 (3)	C17—H17C	0.9600
C5—H5A	0.9300		
			
O1^i^—Cu1—O1	180.0	N1—C10—H10A	118.0
O1^i^—Cu1—O3	87.35 (6)	C2—C10—H10A	118.0
O1—Cu1—O3	92.65 (6)	N1—C11—C12	112.76 (17)
O1^i^—Cu1—O3^i^	92.65 (6)	N1—C11—H11A	109.0
O1—Cu1—O3^i^	87.35 (6)	C12—C11—H11A	109.0
O3—Cu1—O3^i^	180.0	N1—C11—H11B	109.0
C1—O1—Cu1	130.33 (12)	C12—C11—H11B	109.0
C3—O3—Cu1	124.62 (12)	H11A—C11—H11B	107.8
C10—N1—C9	119.95 (15)	C11—C12—H12A	109.5
C10—N1—C11	118.31 (15)	C11—C12—H12B	109.5
C9—N1—C11	121.70 (14)	H12A—C12—H12B	109.5
C7—N2—C13	116.83 (15)	C11—C12—H12C	109.5
C7—N2—C16	117.25 (15)	H12A—C12—H12C	109.5
C13—N2—C16	111.04 (15)	H12B—C12—H12C	109.5
C15—N3—C14	108.25 (16)	N2—C13—C14	108.39 (16)
C15—N3—C17	110.58 (18)	N2—C13—H13A	110.0
C14—N3—C17	110.99 (18)	C14—C13—H13A	110.0
O2—C1—O1	122.66 (17)	N2—C13—H13B	110.0
O2—C1—C2	119.20 (17)	C14—C13—H13B	110.0
O1—C1—C2	118.13 (16)	H13A—C13—H13B	108.4
C10—C2—C3	119.32 (16)	N3—C14—C13	110.51 (17)
C10—C2—C1	116.81 (15)	N3—C14—H14A	109.5
C3—C2—C1	123.84 (16)	C13—C14—H14A	109.5
O3—C3—C2	125.72 (16)	N3—C14—H14B	109.5
O3—C3—C4	118.07 (15)	C13—C14—H14B	109.5
C2—C3—C4	116.19 (15)	H14A—C14—H14B	108.1
C9—C4—C5	118.77 (16)	N3—C15—C16	110.62 (17)
C9—C4—C3	121.23 (16)	N3—C15—H15A	109.5
C5—C4—C3	119.96 (16)	C16—C15—H15A	109.5
C6—C5—C4	119.63 (17)	N3—C15—H15B	109.5
C6—C5—H5A	120.2	C16—C15—H15B	109.5
C4—C5—H5A	120.2	H15A—C15—H15B	108.1
C5—C6—F1	118.45 (17)	N2—C16—C15	109.86 (16)
C5—C6—C7	123.62 (17)	N2—C16—H16A	109.7
F1—C6—C7	117.85 (16)	C15—C16—H16A	109.7
C8—C7—N2	123.85 (16)	N2—C16—H16B	109.7
C8—C7—C6	116.62 (16)	C15—C16—H16B	109.7
N2—C7—C6	119.30 (16)	H16A—C16—H16B	108.2
C7—C8—C9	121.24 (16)	N3—C17—H17A	109.5
C7—C8—H8A	119.4	N3—C17—H17B	109.5
C9—C8—H8A	119.4	H17A—C17—H17B	109.5
N1—C9—C4	118.52 (15)	N3—C17—H17C	109.5
N1—C9—C8	121.36 (15)	H17A—C17—H17C	109.5
C4—C9—C8	120.11 (16)	H17B—C17—H17C	109.5
N1—C10—C2	124.01 (16)		
			
O3—Cu1—O1—C1	−22.51 (19)	C5—C6—C7—N2	−174.06 (19)
O3^i^—Cu1—O1—C1	157.49 (19)	F1—C6—C7—N2	2.6 (3)
O1^i^—Cu1—O3—C3	−160.14 (16)	N2—C7—C8—C9	173.18 (17)
O1—Cu1—O3—C3	19.86 (16)	C6—C7—C8—C9	−1.2 (3)
Cu1—O1—C1—O2	−168.97 (18)	C10—N1—C9—C4	−7.3 (3)
Cu1—O1—C1—C2	12.5 (3)	C11—N1—C9—C4	175.07 (17)
O2—C1—C2—C10	6.8 (3)	C10—N1—C9—C8	171.97 (17)
O1—C1—C2—C10	−174.60 (18)	C11—N1—C9—C8	−5.6 (3)
O2—C1—C2—C3	−171.3 (2)	C5—C4—C9—N1	179.12 (17)
O1—C1—C2—C3	7.3 (3)	C3—C4—C9—N1	1.4 (3)
Cu1—O3—C3—C2	−8.9 (3)	C5—C4—C9—C8	−0.2 (3)
Cu1—O3—C3—C4	172.47 (12)	C3—C4—C9—C8	−177.85 (16)
C10—C2—C3—O3	173.24 (17)	C7—C8—C9—N1	−178.24 (17)
C1—C2—C3—O3	−8.7 (3)	C7—C8—C9—C4	1.0 (3)
C10—C2—C3—C4	−8.1 (3)	C9—N1—C10—C2	5.5 (3)
C1—C2—C3—C4	169.88 (17)	C11—N1—C10—C2	−176.84 (18)
O3—C3—C4—C9	−175.10 (16)	C3—C2—C10—N1	2.7 (3)
C2—C3—C4—C9	6.2 (3)	C1—C2—C10—N1	−175.49 (17)
O3—C3—C4—C5	7.3 (3)	C10—N1—C11—C12	−95.8 (2)
C2—C3—C4—C5	−171.48 (17)	C9—N1—C11—C12	81.9 (2)
C9—C4—C5—C6	−0.4 (3)	C7—N2—C13—C14	−164.32 (17)
C3—C4—C5—C6	177.27 (18)	C16—N2—C13—C14	57.6 (2)
C4—C5—C6—F1	−176.44 (19)	C15—N3—C14—C13	62.2 (2)
C4—C5—C6—C7	0.2 (3)	C17—N3—C14—C13	−176.26 (18)
C13—N2—C7—C8	−15.2 (3)	N2—C13—C14—N3	−60.8 (2)
C16—N2—C7—C8	120.3 (2)	C14—N3—C15—C16	−60.3 (2)
C13—N2—C7—C6	159.03 (19)	C17—N3—C15—C16	178.0 (2)
C16—N2—C7—C6	−65.5 (2)	C7—N2—C16—C15	165.57 (17)
C5—C6—C7—C8	0.6 (3)	C13—N2—C16—C15	−56.5 (2)
F1—C6—C7—C8	177.28 (18)	N3—C15—C16—N2	57.7 (2)

Symmetry codes: (i) −*x*+1, −*y*+1, −*z*+1.
